# The impact of an integrated, interprofessional knowledge translation intervention on access to inpatient rehabilitation for persons with cognitive impairment

**DOI:** 10.1371/journal.pone.0266651

**Published:** 2022-09-01

**Authors:** Elizabeth Linkewich, Jorge Rios, Kay-Ann Allen, Lisa Avery, Deirdre R. Dawson, Michelle Donald, Mary Egan, Anne Hunt, Katelyn Jutzi, Sara McEwen

**Affiliations:** 1 North & East GTA Stroke Network, Sunnybrook Health Sciences Centre, Toronto, Ontario, Canada; 2 Practice-Based Research, Sunnybrook Research Institute, Toronto, Ontario, Canada; 3 Department of Occupational Science and Occupational Therapy, University of Toronto, Toronto, Ontario, Canada; 4 St. John’s Rehab Research Program, Sunnybrook Research Institute, Toronto, Ontario, Canada; 5 Avery Information Services Ltd., Orillia, Ontario, Canada; 6 Rehabilitation Sciences, University of Toronto, Toronto, Ontario, Canada; 7 Rotman Research Institute, Baycrest, Toronto, Ontario, Canada; 8 Central Local Health Integration Network, Markham, Ontario, Canada; 9 School of Rehabilitation Sciences, University of Ottawa, Ottawa, Ontario, Canada; 10 Bloorview Research Institute, Holland Bloorview Kids Rehabilitation Hospital, Toronto, Ontario, Canada; 11 Department of Physical Therapy, University of Toronto, Toronto, Ontario, Canada; Flinders University School of Medicine: Flinders University Medicine, AUSTRALIA

## Abstract

**Introduction:**

Stroke rehabilitation teams’ skills and knowledge in treating persons with cognitive impairment (CI) contribute to their reduced access to inpatient rehabilitation. This study examined stroke inpatient rehabilitation referral acceptance rates for persons with CI before and after the implementation of a multi-faceted integrated knowledge translation (KT) intervention aimed at improving clinicians’ skills in a cognitive-strategy based approach, Cognitive Orientation to daily Occupational Performance (CO-OP), CO-OP KT.

**Methods:**

CO-OP KT was implemented at five inpatient rehabilitation centres, using an interrupted time series design and data from an electronic referral and database system called E-Stroke. CO-OP KT included a 2-day workshop, 4 months of implementation support, health system support, and a sustainability plan. A mixed effects model was used to model monthly acceptance rates for 12 months prior to the intervention and 6 months post.

**Results:**

The dataset was comprised of 2604 pre-intervention referrals and 1354 post. In the mixed effects model, those with CI had a lower pre-intervention acceptance rate than those without. Post-intervention the model showed the acceptance rate for those with CI increased by 8.6% (p = 0.02), whereas those with no CI showed a non-significant increase of less than 1%.

**Conclusions:**

Proportionally more persons with CI gained access to inpatient stroke rehabilitation following an integrated KT intervention.

## Background

Despite evidence of its benefits to them [1], persons with cognitive impairment (CI) following a stroke have reduced access to inpatient rehabilitation [2]. Timely and intensive rehabilitation is important to optimize recovery [3]. While a poorer outcome is generally reported for persons with CI, rehabilitation mitigates this through reducing disability and improving mood and quality of life [4]. Lack of access to inpatient rehabilitation can result in patients being transferred to assisted living facilities without the benefit of rehabilitation that could potentially have enabled return to home. The Toronto Stroke Networks (TSNs) support the implementation of best practice stroke care across the continuum and optimization of resources in Toronto. This large urban city has 12 acute care sites referring to five inpatient stroke rehabilitation programs through an electronic rehabilitation referral system. Approximately 18% of declined referrals to inpatient stroke rehabilitation in Toronto were declined due to CI [5]. In interviews and surveys, rehabilitation team members from all five rehabilitation sites reported a lack of confidence, knowledge, and skills to facilitate recovery in persons with CI [5]. Furthermore, the findings suggested that individual sites all perceived one or more of the other sites were better equipped to support persons with cognitive impairment than were they.

These contributing factors to reduced rehabilitation access are not unique to Toronto. As Ottenbacher and colleagues [6] identified, the attitudes and practice habits of providers can impact access [6]. Longley et al. [7] investigated access specifically for persons with stroke and cognitive impairment, and concluded it is influenced by knowledge gaps of providers and service constraints. They recommend increased training and augmenting patient-centred services. In Toronto, decisions regarding acceptance to rehabilitation are influenced by administrators and the interprofessional care team. Based on this broader evidence and local findings described above, it was postulated that access issues for persons with CI would best be addressed through a knowledge translation (KT) intervention aimed at improving skills and knowledge of providers.

Specifically, we developed, implemented, and evaluated a multi-faceted, supported KT initiative, designed to promote the interprofessional use of the Cognitive Orientation to daily Occupational Performance (CO-OP) [8]. CO-OP is a cognitive strategy-based intervention aligned with Canadian Stroke Best Practice Recommendations for cognitive rehabilitation [9]. It is a functional, patient-goal-centred, problem solving approach that has demonstrated positive effects on function compared to control conditions in persons with stroke [10–13], including those in inpatient rehabilitation with CI [11]. A detailed description of CO-OP’s theoretical foundations, key features, and administration procedures is available in a publication by Polatajko and Mandich, 2004 [8], and adaptations for the stroke population have also been published [14]. The integrated KT intervention, known as CO-OP KT, was implemented in five inpatient stroke rehabilitation programs as outlined in the published protocol [15]. CO-OP KT is fully described below in the methods section; in brief, it includes a 2 day workshop, a 4-month period of implementation support, a consolidation session, infrastructure support and a sustainability plan. The protocol outlined three study questions at the levels of patient, provider, and health system. Patients who attended rehabilitation after the CO-OP KT implementation were significantly more likely to achieve minimally important changes in functional mobility independence than historical controls [16]. Healthcare providers demonstrated and maintained increased CO-OP knowledge and aspects of self-efficacy related to implementing CO-OP, and chart audits provided evidence of practice change [17]. This paper reports on changes at the level of the health system, examining the research question: Is the implementation of CO-OP KT associated with a change in access to inpatient stroke rehabilitation for persons with CI, as indicated by pre and post intervention changes in the proportion of that population accepted to inpatient rehabilitation programs?

## Methods

### Study design

An interrupted time series design was conducted using referral and acceptance data pre and post implementation of the CO-OP KT intervention in five rehabilitation hospitals in Toronto. An interrupted time series design provides an estimate of the effect of an intervention using a series of measurements of dependent variables, divided into pre-intervention and post-intervention segments [18]. It is useful when randomized designs are impossible or impractical. In this case, with only 5 sites, cluster randomization was not possible, thus the interrupted time series was the pragmatic choice. Research Ethics Board approval was obtained at all participating sites.

### Intervention

The CO-OP KT intervention is a supported integrated KT intervention aimed at the interprofessional implementation of CO-OP. It has five elements: a workshop, implementation support, a consolidation session, infrastructure support and a sustainability plan. All aspects of the intervention were co-designed with the knowledge users. All healthcare professionals providing direct care were invited to participate, and the research team encouraged sites to include all or most occupational and physical therapists and speech-language pathologists, at least one nurse, and other interested team members who had a direct client care role. Funds were offered to provide coverage for the team members who attended training. The standardized two-day instructional and practice-based CO-OP *workshop* was followed by a four-month *implementation support* period. During the support period, an implementation facilitator conducted six face-to-face sessions at each site to advance site-specific implementation goals. A CO-OP instructor visited each site once to support ongoing needs, answer questions, and problem-solve specific issues. Physical resources were provided, including a workbook, posters, and information cards. Regular teleconferences were held with administrators overseeing stroke rehabilitation at each site to facilitate organization and system-level support and alignment. A half-day site-specific *consolidation session* was held as a capstone to synthesize learning and plan ongoing implementation. *Infrastructure support* comprised of linking to existing KT resources, such as an established TSNs Virtual Community of Practice (www.strokecommunity.ca) that was utilized as a resource during and after the implementation phase. Following the 4 month implementation support period, a *sustainability plan* was implemented at each site with quarterly TSNs-facilitated site champion meetings and site leadership meetings.

### Variables and data source

In the TSNs, access to rehabilitation is coordinated through a common electronic referral system and database called the E-Stroke Rehabilitation Referral System (E-Stroke). Twelve acute hospital sites refer to five freestanding rehabilitation centres that have expertise in stroke rehabilitation and designated stroke rehabilitation beds. Rehabilitation site representatives review and respond to referrals sent to their site. Data elements extracted from E-Stroke were monthly averages of total referrals and admissions to inpatient stroke rehabilitation, cognitive status, and site.

The cognitive elements memory, attention, judgement, and executive functioning were coded in the E-Stroke database as no impairment; or mild, moderate, or severe impairment. Using the cognitive element with the most severe level of CI, we created a dichotomous variable (no CI or any CI) and a four category variable (no CI, mild CI, moderate CI, and severe CI). Scores on the MoCA© [19] more precise cognitive screening tool, were compared with the dichotomous variable, where a MoCA© was completed (n = 291 referrals). A MoCA© score of > 26 was used to indicate no cognitive impairment. The cognitive status categories demonstrated the following characteristics: sensitivity (97.9%), specificity (62.9%), positive predictive value (85.4%), and negative predictive value (82.1%).

The dataset included all cases for the 12-month period prior to CO-OP KT (October 2015 to September, 2016) and the six month post-intervention period (April 2017 –October 2017). The post-intervention period was limited to 6 months, as the health system put an automatic acceptance policy for persons with moderate stroke in place at that point, impacting future acceptance rates.

The CO-OP KT intervention began with a workshop October 2016, and post-intervention evaluations for all teams were concluded by April 2017. As the research team began direct communications with the stroke rehabilitation team members about the project in the month prior to the workshops, data from this month were also excluded.

### Data analysis

Data were cleaned to include only cases with complete data. *Referral*, rather than individual patient cases, was used as the unit of analysis, as each patient case could have referrals and acceptances to multiple rehabilitation hospitals. Descriptive statistics were conducted to identify the number of referrals with each level of CI (none, mild, moderate, severe) accepted to each site pre and post intervention. The interrupted time series design was analysed using a linear mixed effects model with random site effects. The outcome of interest was the monthly acceptance rate (calculated separately by site), with dichotomous predictors for any cognitive impairment, post-intervention and an interaction term. The interaction term is the estimate of the change in acceptance rate post-intervention for those with CI and is the primary parameter of interest. Random effects were used to model variation across sites and a first order autoregressive term was included to model correlation of repeated measures. The full model with an illustration of the model parameters is provided in the online supplement. The nlme package [20] in the R statistical programming language [21] was used for the mixed effects model, SPSS version 24 was used for all other analyses.

## Results

There were 2,707 referrals to inpatient rehabilitation in the 1-year pre-intervention period and 1,372 in the 6-month follow-up period. For the pre- and post-intervention periods, cognitive status was available for 2,604 and 1,354 referrals respectively. Table 1 provides proportions of acceptances by CI severity for each site, showing considerable variability among sites and categories. Sites 1, 2, and 5 showed overall significant increases in acceptance rates, impacted by increases in those with mild and moderate CI. Site 4 had a non-significant overall acceptance rate decrease (82.6% pre, 77.6% post) likely driven by a significant decrease in acceptance of those with no CI, and Site 3 showed virtually no overall change (85.0% pre, 87.7% post).

**Table 1:** Proportion (%) of referrals accepted by site and cognitive impairment severity.

Site	Cognitive impairment severity								Total	
	None		Mild		Moderate		Severe			
	Pre	Post	Pre	Post	Pre	Post	Pre	Post	Pre	Post
Site 1	86.5	84.1	77.4	84.1	**65.1**	**80.4** *	55.6	40.0	**73.5**	**81.3** *
Site 2	49.3	65.9	**50.0**	**73.7** *	**44.4**	**72.2** *	50.0	62.5	**47.9**	**71.0** *
Site 3	88.4	93.0	86.6	85.8	83.9	87.4	67.9	83.3	85.0	87.7
Site 4	*88*.0*	*63*.*9*	82.4	82.0	81.8	82.4	73.3	71.4	82.6	77.6
Site 5	66.3	74.3	**69.8**	**82.2** *	71.7	75.7	42.3	61.5	**68.7**	**77.1** *
Total	77.1	76.6	75.2	82.1	71.6	80.1	58.9	64.9	73.4	79.5*

*p < .05 based on chi-square analysis. Results in **bold** indicate a significant increase and the result in *italics* indicates a significant decrease.

Table 2 provides the results of mixed effects modeling. Prior to the intervention the baseline acceptance rate was 75.7% and referrals with any CI had an acceptance rate 5.2% lower, on average, than those without (p = .01). The acceptance rate post intervention for persons with CI increased significantly by 8.4% (p = 0.02),compared to the overall acceptance rate that increased less than 1%.

**Table 2:** Mixed effects model examining the effectiveness of the CO-OP KT intervention for increasing acceptance rates for persons with cognitive impairment.

Fixed Effects: Outcome is monthly acceptance rate					
Parameter	Value	SE	df	t	p
Referral rate pre intervention (no CI)	0.757	0.075	182	10.1	< .001
Referral rate pre intervention (CI)	-0.052	0.022	182	-2.4	.018
Post-intervention (no CI)	0.007	0.055	182	0.1	.899
Post-intervention (CI)	0.084	0.036	182	2.4	.020
Random Effect of Rehabilitation Site					
Parameter	SD				
Intercept	0.16				
Intervention	0.10				
Residual	0.15				
Autocorrelation	0.27				

SE = standard error; SD = standard deviation; df = degrees of freedom

## Discussion

This study examined the downstream impacts of a KT study on access to stroke rehabilitation for persons with CI. Results suggest the CO-OP KT intervention was associated with an increased proportion of persons with mild and moderate CI gaining access to inpatient rehabilitation. Controlling for time and site variability, a nearly 9% increase was seen in acceptance for those with any CI compared to those without. Although many factors may have influenced a change in access, significant changes in knowledge, skills, and aspects of self-efficacy of healthcare providers were seen and reported following the CO-OP KT intervention [22], providing evidence that the KT intervention contributed. Below, we elaborate on the potential impact of the KT intervention on patient access and discuss these results in the context of previously reported barriers to access to rehabilitation.

One impetus for implementing CO-OP KT was to change practice habits and attitudes of providers and decision makers who influence access to rehabilitation; increased acceptances to inpatient rehabilitation for persons with mild and moderate CI suggests this may have occurred. Evidence from the CO-OP KT sub-study that examined provider learning outcomes demonstrated improvements in CO-OP knowledge as well as improvements in aspects of self-efficacy and practice [17]. Improvement in front-line providers’ skills and knowledge to treat patients with CI, combined with ongoing engagement of decision-makers, may have shifted attitudes sufficiently to increase the number of patients with CI accepted into rehabilitation.

There were no significant changes in access for persons with severe or no CI. The Evidence Based Review of Stroke Rehabilitation [23], a now outdated Canadian review of best practices, is recorded as one of very few national guidelines that suggests there should be restrictions to rehabilitation for persons with severe stroke [24]. The Canadian Stroke Best Practice Recommendations for Rehabilitation [3] have removed this restriction, but the change may not yet have been fully implemented clinically. The former document has been a longstanding resource for stroke rehabilitation professionals and may still influence stroke rehabilitation professionals’ practice. While the change in the proportion of persons with mild and moderate CI accessing inpatient rehabilitation suggests that attitudes are changing, there is room for more improvement, particularly for persons with severe CI who did not gain more access. It is of note that the CO-OP KT intervention aimed to improve the interprofessional teams’ skills and knowledge to provide a more contemporary, function-based approach to treat a broader range of persons with stroke, *including* persons with CI. The intervention did not differentiate between levels of CI severity. Further KT efforts directed specifically at access for persons with severe CI may be required. While there is evidence suggesting that persons with severe CI can benefit from rehabilitation [25–27] it is limited, and further research aimed at understanding the nuances of rehabilitation for this population is needed.

Site-specific differences in acceptance were present, with three sites showing increased access for persons with mild to moderate CI, one site showing no change, and another showing an overall non-significant decline in acceptances driven by a significant decrease in acceptance in those with *no* CI, which was an aspect of the intended shift. Several factors beyond baseline acceptance rates and the degree of uptake of CO-OP KT could have influenced these differences, particularly differing internal procedures for reviewing and accepting referrals at each stroke rehabilitation program, including how the interprofessional team is involved in the process.

### Limitations

An interrupted time series uses a control condition that is temporally different from the intervention condition, and system changes may occur in the interim. A non-randomized trial with recruitment at 5 sites introduced a high degree of variability, based on site-specific cultural, management, and staffing issues. To mitigate both the temporal and site variability issues, the study had a large number of cases and used a robust mixed effect modeling analysis. All E-Stroke referrals to five rehabilitation centres were included in the study making the results highly representative of large urban cities. This broad implementation study did not allow for monitoring compliance rates; however, results suggest a shift in acceptance despite this.

### Conclusion

The CO-OP KT intervention aimed to promote interprofessional team use of a cognitive strategy-based approach in inpatient stroke rehabilitation. Following CO-OP KT, significantly more persons with CI were admitted to stroke rehabilitation, providing them the potential for greater independence and quality of life. Changes in access varied across the five sites, highlighting that access is a complex construct, and improvement requires a multidimensional approach. This study, together with the other CO-OP KT sub-studies, provides evidence that a multifaceted integrated KT intervention, which included regular engagement with clinicians and administrators, may influence attitudinal barriers to access. A future KT intervention targeted at changing knowledge and attitudes about the benefits of rehabilitation for persons with severe CI after stroke may shift access further.

## Supporting information

S1 Dataset (CSV)
